# The effects of larval organic fertiliser exposure on the larval development, adult longevity and insecticide tolerance of zoophilic members of the *Anopheles gambiae* complex (Diptera: Culicidae)

**DOI:** 10.1371/journal.pone.0215552

**Published:** 2019-04-18

**Authors:** Alexander C. S. N. Jeanrenaud, Basil D. Brooke, Shüné V. Oliver

**Affiliations:** 1 Centre for Emerging Zoonotic and Parasitic Diseases, National Institute for Communicable Diseases of the National Health Laboratory Service, Johannesburg, South Africa; 2 Wits Research Institute for Malaria, MRC Collaborating Centre for Multi-disciplinary Research on Malaria, School of Pathology, Faculty of Health Sciences, University of the Witwatersrand, Johannesburg, South Africa; University of Queensland, AUSTRALIA

## Abstract

Zoophilic members of the *Anopheles gambiae* complex are often associated with cattle. As such, it is likely that the immature aquatic stages will be exposed to cattle faeces as a pollutant. This study aimed to examine the effect of cattle manure on members of the *An*. *gambiae* complex found in South Africa. In this study, a commercial organic fertiliser originating from cattle manure was used as a proxy for cattle faeces. Laboratory strains of *An*. *merus*, *An*. *quadriannulatus* as well as four *An*. *arabiensis* strains (SENN and MBN: insecticide susceptible, MBN-DDT: insecticide resistant, unselected, SENN-DDT: insecticide resistant: selected for resistance) were used in this study. The effect of larval fertiliser exposure on larval development rate and adult longevity was assessed in all three species. The effect of larval fertiliser exposure on subsequent adult size, insecticide tolerance and detoxification enzyme activity of the four strains of the malaria vector *An*. *arabiensis* was also assessed. Following fertiliser treatment, all strains and species showed a significantly increased rate of larval development, with insecticide susceptible strains gaining the greatest advantage. The adult longevities of *An*. *merus*, *An*. *quadriannulatus*, insecticide susceptible and resistant *An*. *arabiensis* were significantly increased following fertiliser treatment. Insecticide susceptible and resistant *An*. *arabiensis* adults were significantly larger after larval organic fertiliser exposure. Larval fertiliser exposure also increased insecticide tolerance in adult *An*. *arabiensis*, particularly in the insecticide resistant, selected strain. This 4.7 fold increase in deltamethrin tolerance translated to an increase in pyrethroid resistance intensity, which could exert operational effects. In general, larval exposure to cattle faeces significantly affects the life histories of members of the *An*. *gambiae* complex.

## Introduction

The *Anopheles gambiae* complex consists of eight morphologically indistinguishable species that differ in their behaviour and malaria vectorial capacity [1, 2]. Despite these differences, their larval environments are generally similar. All member species of the complex breed in temporary bodies of water that are usually sunlit, shallow and clean [2, 3]. This however appears to be changing as members of the complex, the major malaria vector species *An*. *gambiae* and *An*. *arabiensis* in particular, will also breed in polluted water [4, 5]. This has numerous effects on their life histories and, most importantly, selection for resistance to insecticides [6, 7].

The role of pollution as a mode of selection for insecticide resistance is well examined—see review by [8]. When examining pollution, however, there is a tendency to examine anthropogenic pollutants, usually toxic in nature, such as heavy metals or agrochemicals such as herbicides [9, 10]. Even more innocuous pollutants such as inorganic fertilizers can also affect life history [11, 12]. Very little attention has so far been paid to organic pollutants, despite the close relationship between cattle and the *An*. *gambiae* complex, with the relationship of *An*. *arabiensis*, as well as other zoophilic members,and cattle particularly important.

*An*. *arabiensis* is partially characterised by its behavioural plasticity, and is generally described as exophagic and exophilic [3]. *An*. *arabiensis* are zoophilic nocturnal feeders but are also opportunistic feeders, particularly of humans. Importantly, this species is known to adopt both endophagic and endophilic behavioural patterns if potential hosts primarily reside indoors [13]. This behavioural plasticity means that this species is not easily controlled by traditional methods that are based on the indoor deployment of insecticides [14], leading to ongoing residual transmission despite control interventions [15]. *An*. *arabiensis* is a primary vector of malaria in South Africa [16], and resistance to both DDT and pyrethroids have been reported in KwaZulu-Natal, one of the country’s malarious provinces [17, 18]. This species therefore represents a threat to South Africa’s malaria elimination agenda.

*An*. *arabiensis* tends to flourish in agricultural regions. As such, the relationship between this species and the agricultural and urban industry is an important one [19–21]. Additionally, *An*. *arabiensis* tends to flourish in association with maize and rice farming [21–23]. This species is especially closely associated with cattle as a primary blood source [24, 25] and is therefore highly likely to be exposed to cow dung that pollutes breeding sites such as rice paddies [26]. Within the *An*. *gambiae* complex, the non-vector *An*. *quadriannulatus* and the minor malaria vector *An*. *merus* are often found breeding in the same habitats as the major vector *An*. *arabiensis*. *An*. *quadriannulatus* has never been implicated in malaria transmission and is an endophilic, endophagic and zoophilic species [3]. DDT resistance has been reported in South African *An*. *quadriannulatus* [17]. *Anopheles merus* is a localised malaria vector, and this exophilic, exophagic species is notable for being capable of breeding in sites with a salt content that is usually not tolerable to other mosquitoes [2, 3]. In districts of Mpumalanga Province, South Africa, increasing numbers of *An*. *merus* and *An*. *quadriannulatus* are being detected [27]. The larval dynamics of sympatric species are important, as they could have potential effects on malaria transmission dynamics [28, 29]

The presence of inorganic pollutants appear to favour the development of insecticide resistance [30], and insecticide tolerant larvae are at an advantage in polluted conditions [31, 32], but usually at a biological cost [33]. For inorganic pollutants and various organic agrochemicals this is not surprising, as their relatively late introduction into the environment means that mosquitoes would not have evolved to cope with them as they would have phytochemicals. It must be noted that in some pollutants, notably the herbicide glyphosate, hormetic effects have been noted [34, 35]. Hormesis is the phenomenon whereby an exposure compound may confer fitness advantages or disadvantages depending on the dose (reviewed in [34]). Organic water body pollutants such as cattle faeces are not regulated as are other water pollutants, as they are non-toxic.

Despite the close association between agricultural activities, the presence of cattle and the incidence of malaria, very little information is available concerning the role of cattle waste on malaria vector life histories. A single study reported an increase in both *Anopheles* and *Culex* larvae breeding in sewage-contaminated water, hypothesising that the faecal matter increased the nutrient content of the water, resulting in increased larval density. High culicine density was accompanied by low anopheline density [36]. Another study noted that increased organic waste increased the efficacy of methoprene for larval control [37]. The aim of this project was therefore to examine the effect of organic pollutants on the life histories of certain member species of the *An*. *gambiae* complex, with a special focus on the effect on *An*. *arabiensis*.

## Materials and methods

### Materials

All *Anopheles* mosquito strains used in this project were housed in the Botha de Meillon insectary and reared as described in [38]. In brief, larvae were reared in reverse osmosis water at 25°C (±2°C) and 80% relative humidity (±5%) with a 12 hour light/dark cycle with a 30 minute dusk/dawn cycle. Larvae were fed a mixture of powder Beano dog biscuits and yeast.

#### Anopheles arabiensis

SENN: An insecticide susceptible strain, originating in Sennar, Sudan. It has been in colony since 1980.

SENN-DDT: An insecticide resistant strain selected from SENN, and has been continuously selected for DDT resistance. It is resistant to multiple insecticides, is fixed for the *kdr* L1014F mutation and has elevated detoxification enzyme levels [39–41].

MBN: An insecticide susceptible strain from KwaZulu-Natal Province, South Africa.

MBN-DDT: An insecticide resistant strain selected for DDT resistance. This strain is not currently under selection, but still displays resistance [42]. The basis for resistance in this strain is primarily metabolic [43].

#### Anopheles quadriannulatus

SANGWE: An insecticide susceptible strain originating from Zimbabwe.

#### Anopheles merus

MAFUS: AN insecticide susceptible strain, established in 2012 and originating from Mpumalanga Province, South Africa. The strain is reared in 8.5% w/v salt water.

### Methods

The commercial organic fertiliser (OF) Fertilis was used as a proxy for cattle faeces (Fertilis-Fertiliser made from dairy cow manure processed by earthworms; reg number: B3664 Act 36/1947; Planner Bee Plant care, Kyalamyi, South Africa). An attempt was made to determine a lethal dose, but the fertiliser was found to be non-toxic as no significant mortality could be determined. Therefore, for the sake of water clarity, the dose for all treatments was set at 0.5% (V:V).

### The effect of larval OF exposure on development time

For each strain, 100 first instar larvae (less than 24 hours old) were exposed to OF-polluted water. Larvae reared in clean water served as a control. All treatments were fed the same amount of food and were maintained under standard insectary conditions. The time to pupation was monitored. The experiment was replicated three times with larvae originating from three different egg batches per strain.

### The effect of larval OF exposure on adult longevity

Samples of larvae from each strain were reared while exposed to OF (except for the controls) as described for the development experiment. Pupae were collected and the adults were allowed to emerge. Thirty males and females of each strain were collected and their longevity was monitored until death. Adults were allowed *ad libitum* access to a 10% sucrose solution, and the females were not offered blood meals. Cadavers were removed daily. The experiment was replicated in triplicate.

### The effect of larval OF exposure on *An*. *arabiensis* adult size

Samples of larvae from the insecticide susceptible *An*. *arabiensis* strains SENN and MBN, as well as the insecticide resistant SENN-DDT and MBN-DDT were reared in OF-treated water as described for the development experiment. Thirty males and females of each of strain were cold-killed and their wings were removed. Wing length was measured using an Olympus SZX7. Wing lengths were measured from wing tip to allula and used as a proxy for adult size [44]. The sizes of OF-treated adults were compared to those of adults reared in clean water.

### The effect of larval OF exposure on *An*. *arabiensis* adult insecticide lethal time (LT50)

Samples of larvae from the insecticide susceptible *An*. *arabiensis* strains SENN and MBN, as well as the insecticide resistant SENN-DDT and MBN-DDT were reared in OF-treated water as described for the development experiment. Three days old, non-blood fed females and males of equivalent age were exposed to discriminating concentrations of malathion and deltamethrin using CDC bottle bioassays to determine the lethal time to 50% mortality (LT50). The insecticide susceptibility assays are described in [32]. In brief, a dosage of 10μg/ml and 1μg/ml of either malathion or deltamethrin was used to coat bottles for SENN-DDT and SENN respectively. A range of exposure times were used to determine the respective LT50s: 2, 4, 8, 16 and 32 minutes for SENN and 10, 20, 40 and 80 minutes for SENN DDT. The varying exposure times and concentrations were chosen to compensate for the differences in insecticide susceptibility between strains. The experiment was replicated three times with larvae originating from three different egg batches per strain.

### The effect of larval OF exposure on *An*. *arabiensis* adult insecticide resistance intensity

Due to the effect of larval exposure to OF on subsequent SENN-DDT adult insecticide tolerance, the effect of OF exposure was also examined on pyrethroid resistance intensity [45]. SENN DDT first instar larvae were reared in OF-polluted water, with larvae reared in clean water as a control. Pyrethroid resistance intensity was determined as described in [42]. The experiment was replicated three times with larvae originating from three different egg batches per strain.

### The effect of larval OF exposure on *An*. *arabiensis* adult detoxification enzyme activity

SENN, SENN-DDT, MBN and MBN-DDT larvae were reared in OF-treated water, with control larvae reared in clean water. All treatments were fed the same amount of larval food. Adults were subsequently harvested and 48 males and females of from all treatments were cold-killed at the age of three days after access to sucrose only. These adults were then homogenised in PCR-grade water. Cytochrome P450 activity, measured as haeme peroxidase activity, Glutathione S-transferase activity, general esterase activity and catalase activity was assessed calorimetrically as described in [31].

### Statistical analysis

Data were analysed using Statistix 8 (Analytical Software, Tallahassee, Florida) and IBM SPSS statistics version 22 (IBM Corp. Released 2013. IBM SPSS Statistics for Windows, Version 22.0. Armonk, NY). Data were analysed for normality using the Shapiro-Wilk test. Differences in normally-distributed data were analysed using 1-way Analysis of Variance or 2-sample t-test. Non-parametric data were analysed using a Kruskal-Wallis one-way non-parametric analysis of variance. Longevity was assessed using the Kaplan-Meier estimator, with the Log Rank test used as a measure of significance. Data were analysed at a confidence interval of 95%.

## Results

### The effect of larval OF exposure on development time

**Fig 1:**
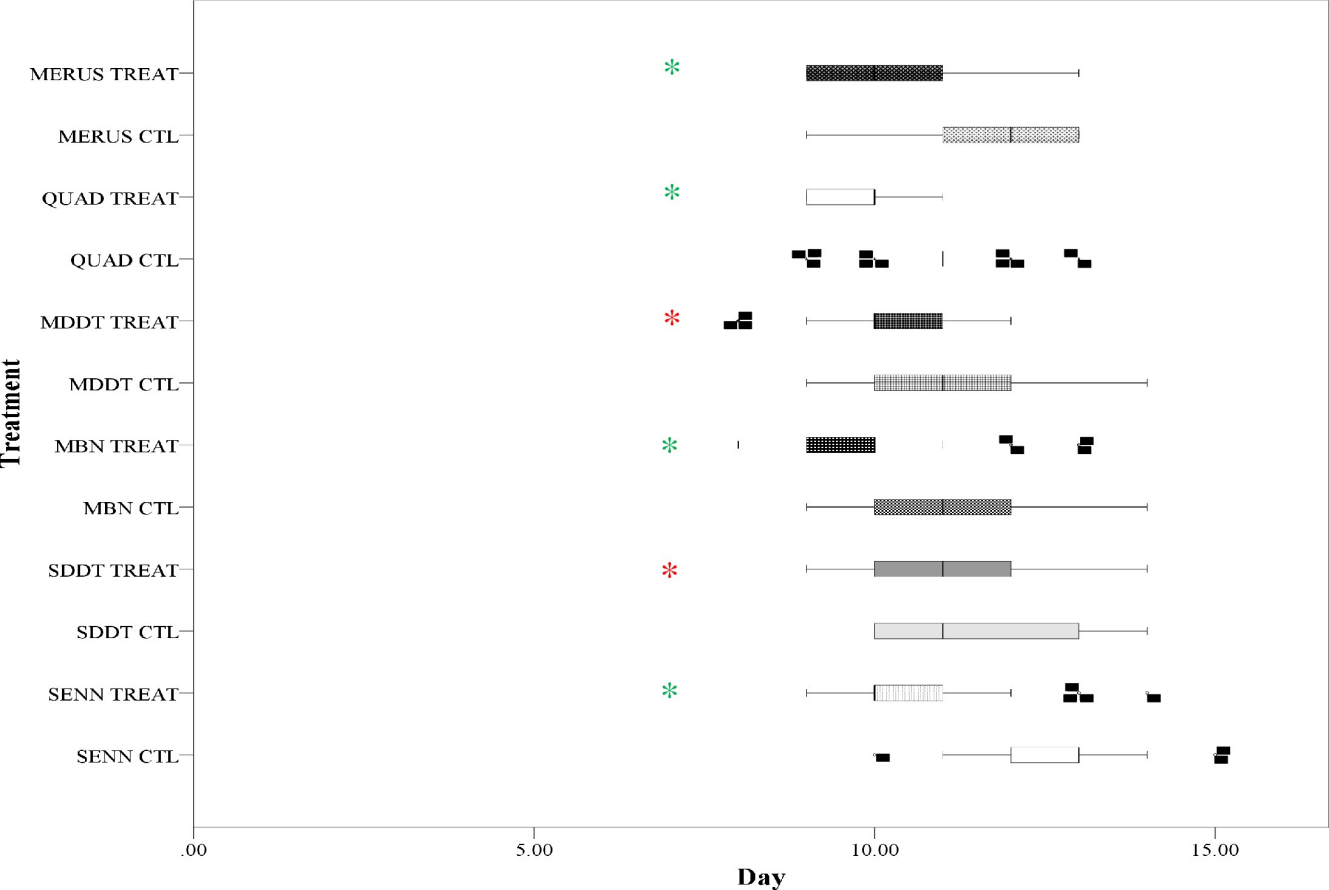
The effect of organic fertiliser exposure on the larval development of members of the *Anopheles gambiae* complex. Larval exposure to organic fertiliser results in a significant increase in time to pupation in all members of the *An*. *gambiae* complex. Asterisks indicate a significant change from the control, with green asterisks indicating a change in insecticide susceptible strains and red asterisks indicating a change in insecticide resistant strains.

Larval OF exposure significantly decreased the time to pupation in all strains (Kruskal Wallis non parametric ANOVA: p<0.01, F = 69.6, DF = 11). The advantages gained by the susceptible *An*. *arabiensis* strains were greater than that of the resistant strains (Kruskal Wallis non parametric ANOVA:SENN vs SENN-DDT: p = 0.04, F = 4.06, DF = 1; MBN vs MBN-DDT: P<0.01, F = 10.8, DF = 1). There was a significant difference in development rate between the SENN and SENN DDT strains under control conditions (Kruskal Wallis non parametric ANOVA: p<0.01, F = 29.3, DF = 1) and treated conditions (Kruskal Wallis non parametric ANOVA: p = 0.04, F = 4.06, DF = 1). MBN and MBN-DDT developed at the same rate under control conditions (Kruskal Wallis non parametric ANOVA: p = 0.11, F = 2.45, DF = 1), but MBN developed significantly faster under OF-treated conditions (Kruskal Wallis non parametric ANOVA: p<0.01, F = 10.9, DF = 1) (Fig 1).

### The effect of larval OF exposure on adult longevity

**Fig 2:**
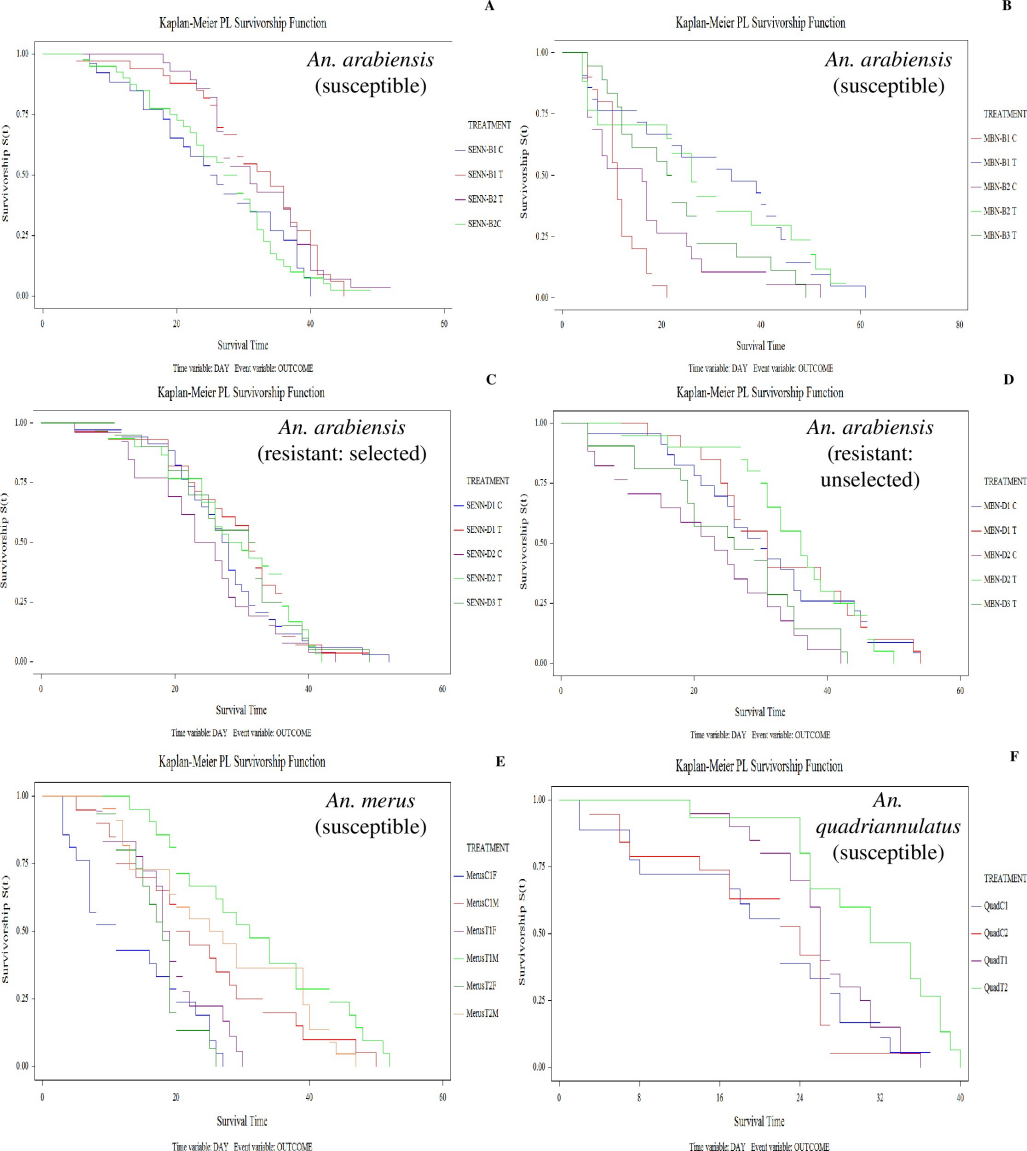
The effect of larval organic fertiliser exposure on the adult longevity of *Anopheles gambiae* complex members. A: The effect of larval organic fertiliser (OF) exposure on the adult longevity of the SENN (*An*. *arabiensis*; insecticide susceptible) strain. B: The effect of larval OF exposure on the adult longevity of the MBN (*An*. *arabiensis*: insecticide susceptible) strain. C: The effect of larval OF exposure on the adult longevity of the SENN-DDT (*An*. *arabiensis*: insecticide resistant, selected) strain. D: The effect of larval OF exposure on the adult longevity of the MBN-DDT (*An*. *arabiensis*: insecticide resistant, unselected) strain. E: The effect of larval OF exposure on the adult longevity of the *An*. *merus* strain, MAFUS (insecticide susceptible). F: The effect of larval OF exposure on the adult longevity of the *An*. *quadriannulatus* strain, SANGWE (insecticide susceptible).

Adults of the insecticide susceptible strain SENN lived significantly longer when the larvae were exposed to OF (Log rank test: p = 0.04, χ^2^ = 8.32, DF = 4) (Fig 2A). This was also true for the susceptible MBN strain (Log rank test: p<0.01, χ^2^ = 16.26, DF = 4) (Fig 2B). There was no significant difference in adult longevity in SENN-DDT after larval OF exposure (Fig 2C). The MBN-DDT strain also showed a significant increase in adult longevity after larval OF exposure (Log rank test: p = 0.02, χ^2^ = 11.32, DF = 4)(Fig 2D).

*Anopheles merus* males but not females showed a significant increase in longevity after larval OF exposure (Log rank test: female: p = 0.17, χ^2^ = 5.05, DF = 4; male: p = 0.02, χ^2^ = 11.51, DF = 4) (Fig 2D). A significant increase in adult longevity was also observed in OF-treated *An*. *quadriannulatus* (Log rank test: female: p<0.01, χ^2^ = 17.66, DF = 4; male: p<0.01, χ^2^ = 15.71, DF = 4) (Fig 2E).

### The effect of larval OF exposure on *An*. *arabiensis* adult size

**Fig 3:**
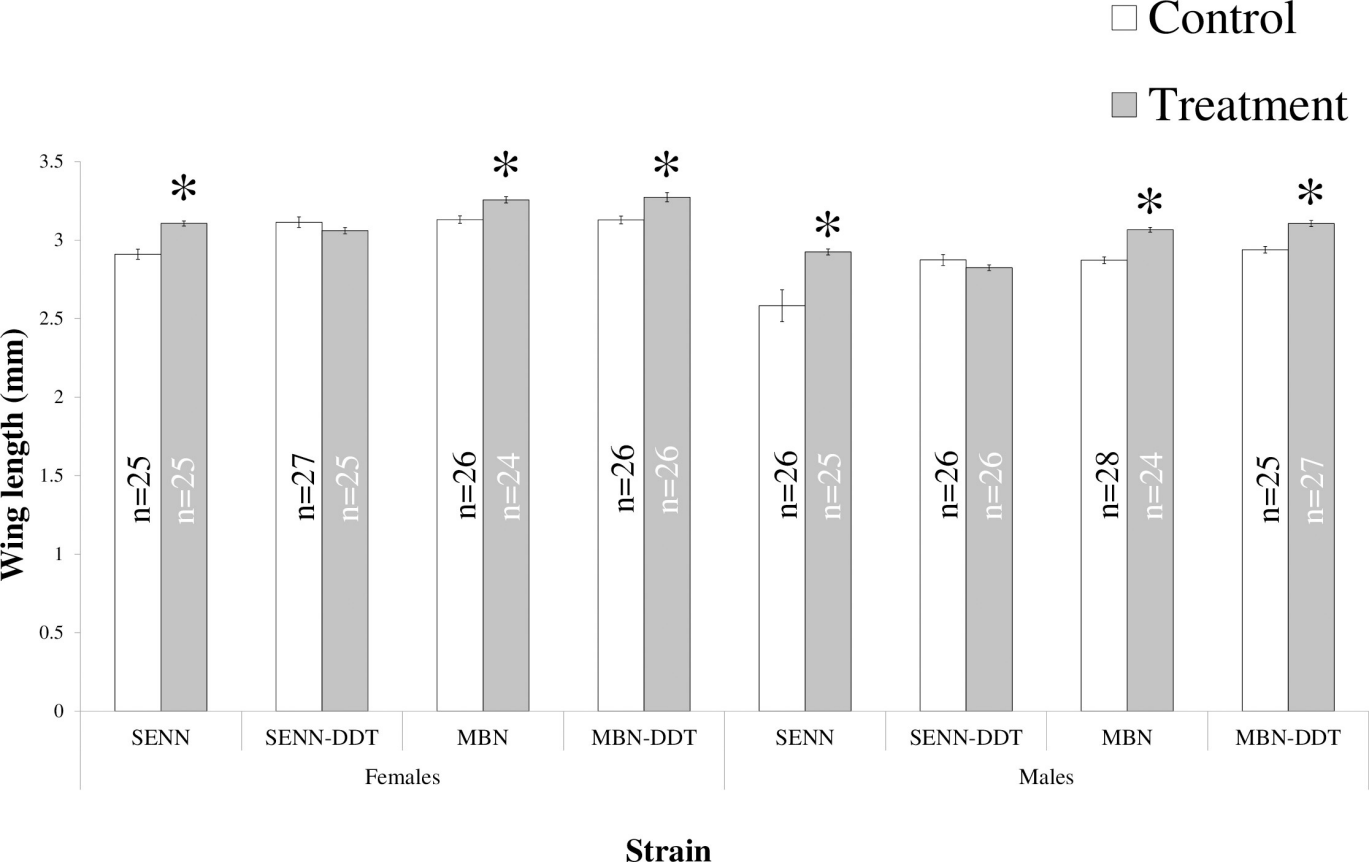
The effect of larval organic fertiliser exposure on subsequent adult size in the malaria vector *Anopheles arabiensis*. Wing length was used a proxy for size. Asterisks indicate a significant change from the controls.

Larval OF exposure resulted in a significant increase in adult size of the susceptible SENN strain for males (2-sample t-test: p<0.01, t = -3.26, DF = 49) and females (2-sample t-test: p<0.01, t = -5.35, DF = 48). This was also true for MBN males (2-sample t-test: p<0.01, t = -7.42, DF = 50) and females (2-sample t-test: p<0.01, t = -3.26, DF = 49). The insecticide resistant strain MBN-DDT also showed an increase in adult size for males (2-sample t-test: p<0.01, t = -5.79, DF = 49.2) and females (2-sample t-test: p<0.01, t = -3.75, DF = 48.5) in association with OF treatment at the larval stage. By contrast, no difference in adult size was observed after OF larval treatment in the insecticide resistant SENN DDT strain for either males (2-sample t-test: p = 0.21, t = 1.25, DF = 50) or females (2-sample t-test: p = 0.17, t = 1.39, DF = 50). SENN males had the greatest fold increase (1.13 fold) in association with OF treatment at the larval stage (Fig 3).

### The effect of larval OF exposure on *An*. *arabiensis* adult insecticide lethal time

**Fig 4:**
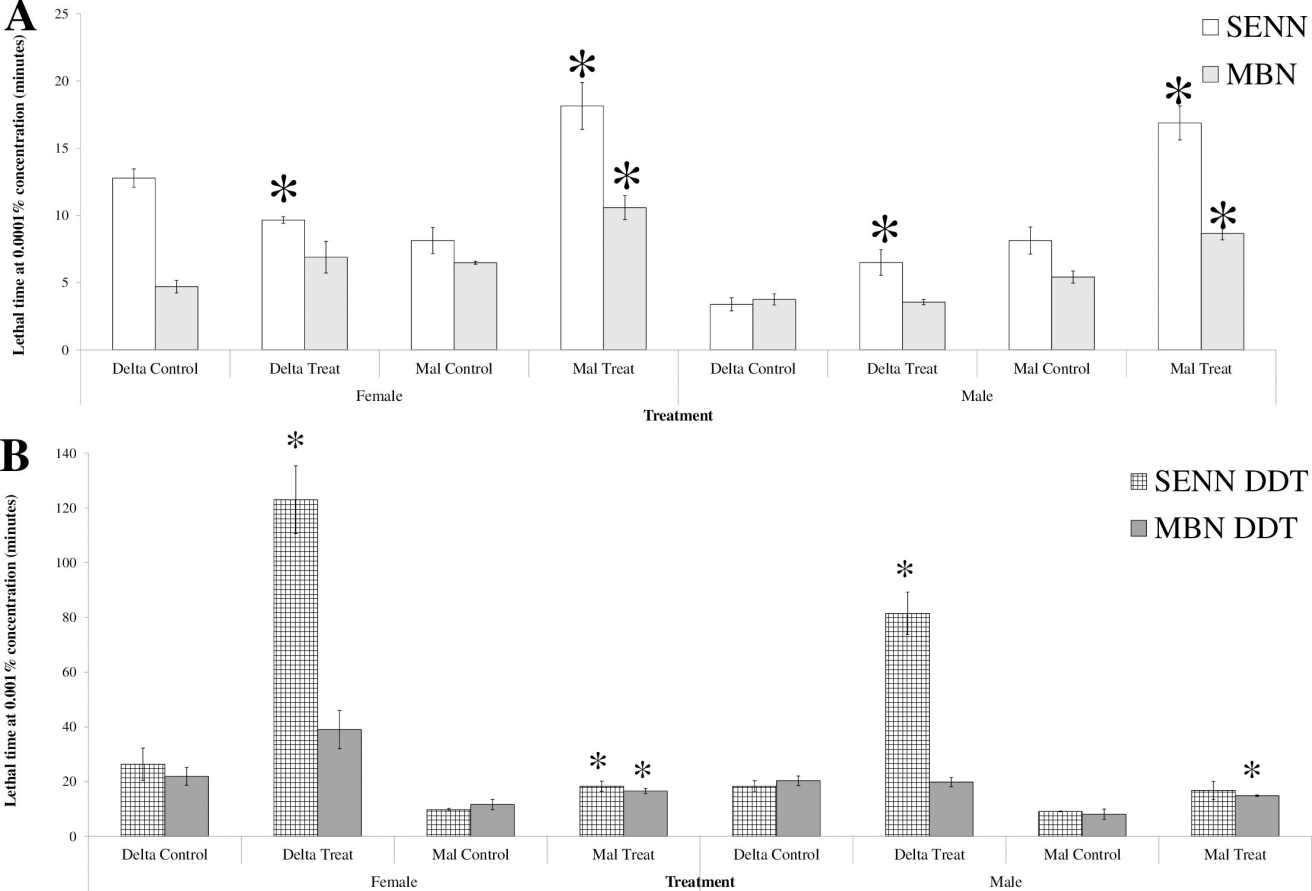
The effect of larval organic fertiliser exposure on the adult lethal time in the malaria vector *Anopheles arabiensis*. A: LT50s of insecticide susceptible *An*. *arabiensis*. B: LT50s of insecticide resistant *An*. *arabiensis*. Asterisks indicate a significant change from the controls.

For the insecticide susceptible strain SENN, OF treatment resulted in a significant decrease in deltamethrin LT50 (1-way ANOVA: p = 0.01, F = 18.6, DF = 1), but an increase in LT50 in males (1-way ANOVA: p = 0.03, F = 8.52, DF = 1). Treatment did not result in a significant change in deltamethrin LT50 for either males (1-way ANOVA: p = 0.68, F = 0.19, DF = 1) or females (1-way ANOVA: p = 0.13, F = 3.00, DF = 1). Larval OF treatment increased malathion LT50 for both SENN (1-way ANOVA: p<0.01, F = 33.9 DF = 3, Tukey’s critical value for comparison: 5.11) and MBN (1-way ANOVA: p<0.01, F = 17.2, DF = 3, Tukey’s critical value for comparison: 2.33) (Fig 4A).

A similar pattern was observed for malathion resistance in SENN-DDT and MBN-DDT females, where larval OF treatment resulted in a significant increase in malathion LT50 (1-way ANOVA: SENN-DDT: p<0.01, F = 19.7, DF = 1; MBN-DDT p<0.01, F = 17.9, DF = 1). For males, a significant increase in malathion LT50 was observed for MBN-DDT (1-way ANOVA: p = 0.03, F = 7.54, DF = 1), but not SENN-DDT (1-way ANOVA: p = 0.06, F = 5.30, DF = 1). Deltamethrin LT50 was not affected in either MBN-DDT males after treatment (1-way ANOVA: p = 0.85, F = 0.04, DF = 1), or in females (1-way ANOVA: p = 0.07, F = 4.92, DF = 1). By contrast, larval OF treatment resulted in a significant increase in deltamethrin LT50 in SENN-DDT males and females (1-way ANOVA: p<0.01, F = 38.4, DF = 3, Tukey’s critical value for comparison: 33.4) (Fig 4B).

### The effect of larval OF exposure on *An*. *arabiensis* adult insecticide resistance intensity

SENN DDT adults had the longest pyrethroid lethal time, and were therefore used to determine whether this increase in tolerance resulted in an increase in pyrethroid resistance intensity. Untreated SENN DDT showed a moderate resistance intensity to permethrin, but this was increased to high after larval OF treatment in both males and females. Similarly, SENN DDT had a low resistance intensity to deltamethrin (as defined in [42]), but after OF treatment resistance intensity increased to high (Table 1).

**Table 1:** The effect of larval organic fertiliser exposure on insecticide resistance intensity in subsequent *Anopheles arabiensis* adults by strain.

Strain	Overall % mortality (*n*; no. replicates)			Resistance intensity	
	DC	5× DC	10× DC		Insecticide
SENN DDT female	67 (118;4)	100 (103; 4)	–	low	Deltamethrin
SENN DDT male	92(100;4)	100 (106;4)	–	low	Deltamethrin
OF Males	39 (100;4)	92 (184; 9)	96(146;6)	high	Deltamethrin
OF females	21(106;4)	49 (213;15)	77(214;9)	high	Deltamethrin
SENN DDT female	30 (100; 4)	89.04 (146; 6)	100 (104; 4)	moderate	Permethrin
SENN DDT male	36(104;4)	92.5(120; 5)	100(124;5)	moderate	Permethrin
OF Males	39 (100;4)	47 (216; 9)	82(140;5)	high	Permethrin
OF Females	21(106;4)	25 (219;15)	77(48;2)	high	Permethrin

DC = discriminating concentration. OF = organic fertiliser treatments. Resistance intensity scored according to [39].

### The effect of larval OF exposure on *An*. *arabiensis* adult detoxification enzyme activity

**Fig 5:**
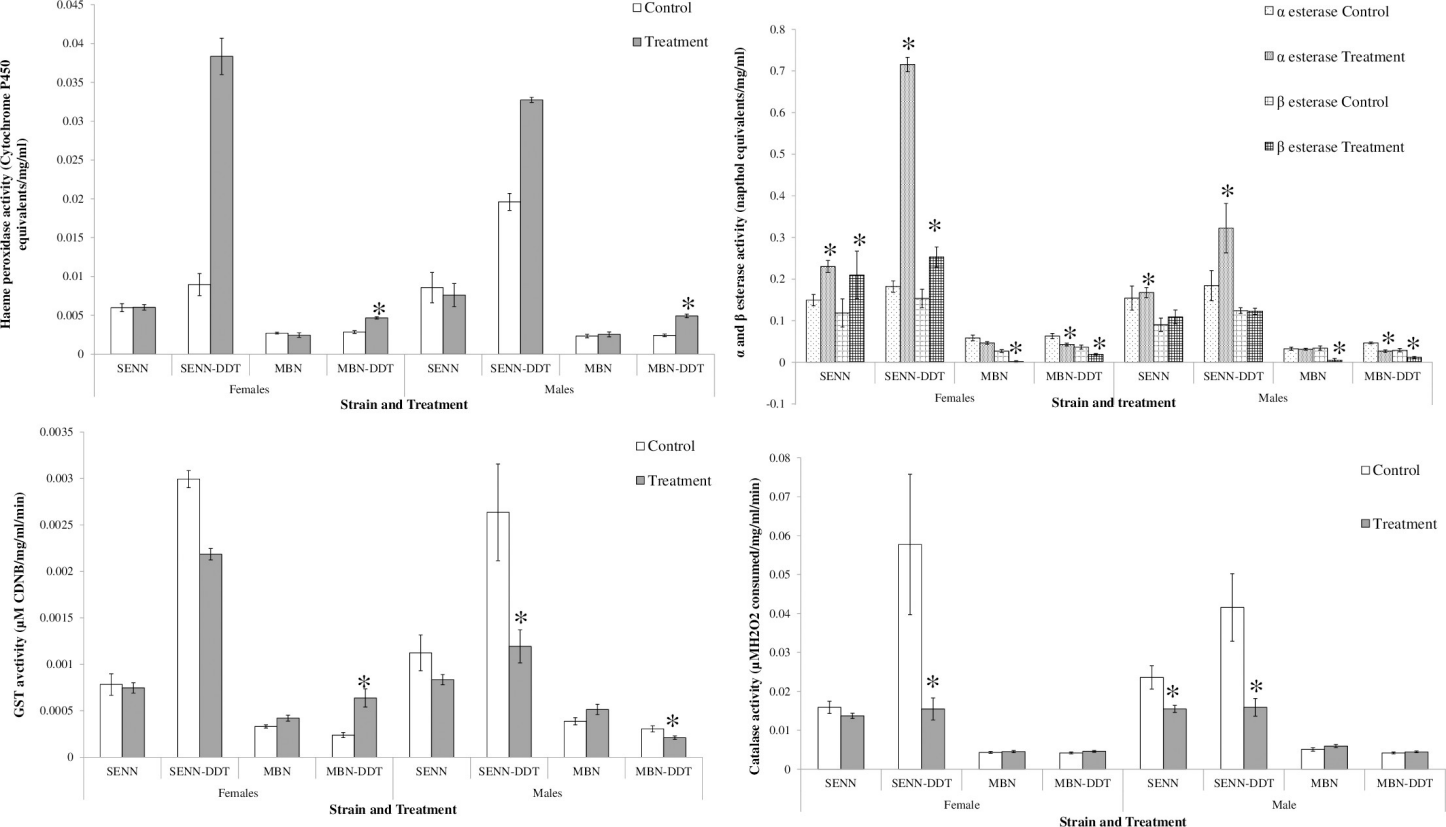
The effect of larval organic fertiliser exposure on adult detoxification and oxidative stress enzyme activity in the malaria vector *Anopheles arabiensis*. A: Haeme peroxidase activity. Haeme peroxidase activity was used as a proxy for cytochrome P450 activity. B: General esterase activity. C: Glutathione S-transferase (GST) activity. D: Catalase activity. Significant differences to the controls of the same strain and sex are indicated by an asterisk.

Cytochrome P450 activity was assessed using haeme peroxidase activity as a proxy. No changes in P450 activity were observed in MBN females (Kruskal-Wallis one-way AOV: p = 0.37, F = 0.82, DF = 1) or males (Kruskal-Wallis one-way AOV: p = 0.59, F = 0.29, DF = 1) in association with OF treatment. MBN-DDT females showed a significant increase in activity post OF treatment (Kruskal-Wallis one-way AOV: p<0.01, F = 43.3, DF = 1) as did MBN-DDT males (Kruskal-Wallis one-way AOV: p<0.01, F = 47.1, DF = 1). SENN females did not show a significant change in P450 activity post OF treatment (Kruskal-Wallis one-way AOV: p = 0.58, F = 0.31, DF = 1) and neither did SENN males (Kruskal-Wallis one-way AOV: p = 0.41, F = 0.68, DF = 1). SENN-DDT males and females showed no significant increase in activity post OF treatment (Kruskal-Wallis one-way AOV: p = 0.14, F = 2.23, DF = 1; males: p = 0.9, F = 0.01, DF = 1)(Fig 5A).

No differences were observed after OF treatment for α-esterase activity in MBN (Kruskal-Wallis One-way AOV: females: p = 0.82, F = 0.05, DF = 1; p = 0.86, F = 0.03, DF = 1). MBN-DDT females and males had significantly reduced α-esterase activities (Kruskal-Wallis One-way AOV: females: p = 0.01, F = 6.96, DF = 1; males: p<0.01, F = 29.1, DF = 1) in association with OF treatment. SENN males and females had a significant increase in α-esterase activity (Kruskal-Wallis One-way AOV: females: p<0.01, F = 23.2, DF = 1; males: p = 0.03, F = 4.78, DF = 1). The same pattern was observed in SENN-DDT, with increased α-esterase activity in males and females (Kruskal-Wallis One-way AOV: females: p = 0.02, F = 6.17, DF = 1; males: p = 0.03, F = 4.77, DF = 1) (Fig 5B).

MBN males and females had significantly reduced β-esterase activity after OF treatment (Kruskal-Wallis One-way AOV: females: p<0.01, F = 51.8, DF = 1; males: p = 0.02, F = 4.78, DF = 1). Similarly, MBN-DDT females had a similar decrease in β-esterase activity after OF treatment (Kruskal-Wallis One-way AOV: females: p<0.01, F = 27.3, DF = 1; males: p<0.01, F = 29.6, DF = 1). SENN females had a significant increase in β-esterase activity (Kruskal-Wallis One-way AOV: p<0.01, F = 87.5, DF = 1) but males did not display any changes in activity (Kruska;-Wallis One-way AOV: p = 0.26, F = 1.30, DF = 1) in association with OF treatment. SENN-DDT females had a significant increase in activity post OF treatment (Kruskal-Wallis One-way AOV: p<0.01, F = 13.5, DF = 1), but the same was not observed in males (Kruskal-Wallis One-way AOV: p = 0.79, F = 0.08, DF = 1).

No differences were observed after OF treatment for GST activity in MBN (Kruskal-Wallis One-way AOV: Females: p = 0.06, F = 3.81, DF = 1; Males: p = 0.05, F = 4.00, DF = 1). MBN-DDT females had a significantly increased GST activity (Kruskal-Wallis One-way AOV: p<0.01, F = 17.1, DF = 1), while males had significantly decreased GST activity (Kruskal-Wallis One-way AOV: p = 0.02, F = 5.84, DF = 1) in association with OF treatment. SENN had no significant changes after OF treatment (Kruskal-Wallis One-way AOV: Females: p = 0.44, F = 0.59, DF = 1;p = 0.55, DF = 0.35, DF = 1). No changes were observed in SENN-DDT GST activity post OF treatment in females (Kruskal-Wallis One-way AOV: p = 0.54, F = 0.38, DF = 1), but resulted in a significant decrease in males (Kruskal-Wallis One-way AOV: p = 0.0.1, F = 7.15, DF = 1) (Fig 5C).

No differences were observed after OF treatment for catalase activity in MBN (1-way ANOVA: Females: p = 0.54, F = 0.36, DF = 1; Males: p = 0.16, F = 2.02, DF = 1). The same was true for MBN-DDT (1-way ANOVA: Females: p = 0.25, F = 1.36, DF = 1; Males: p = 0.44, F = 0.61, DF = 1). The same was also true for SENN females (1-way ANOVA: p = 0.20, F = 1.71, DF = 1) but a significant decrease was observed for SENN males: (1-way ANOVA: p = 0.01, F = 6.70, DF = 1) in association with OF treatment. SENN-DDT males and females had a significantly reduced catalase activity after treatment (1-way ANOVA: p = 0.0.1, F = 3.81, DF = 3, Tukey’s critical Q = 3.71) (Fig 5D).

### Consumption of organic fertiliser by larvae

**Fig 6:**
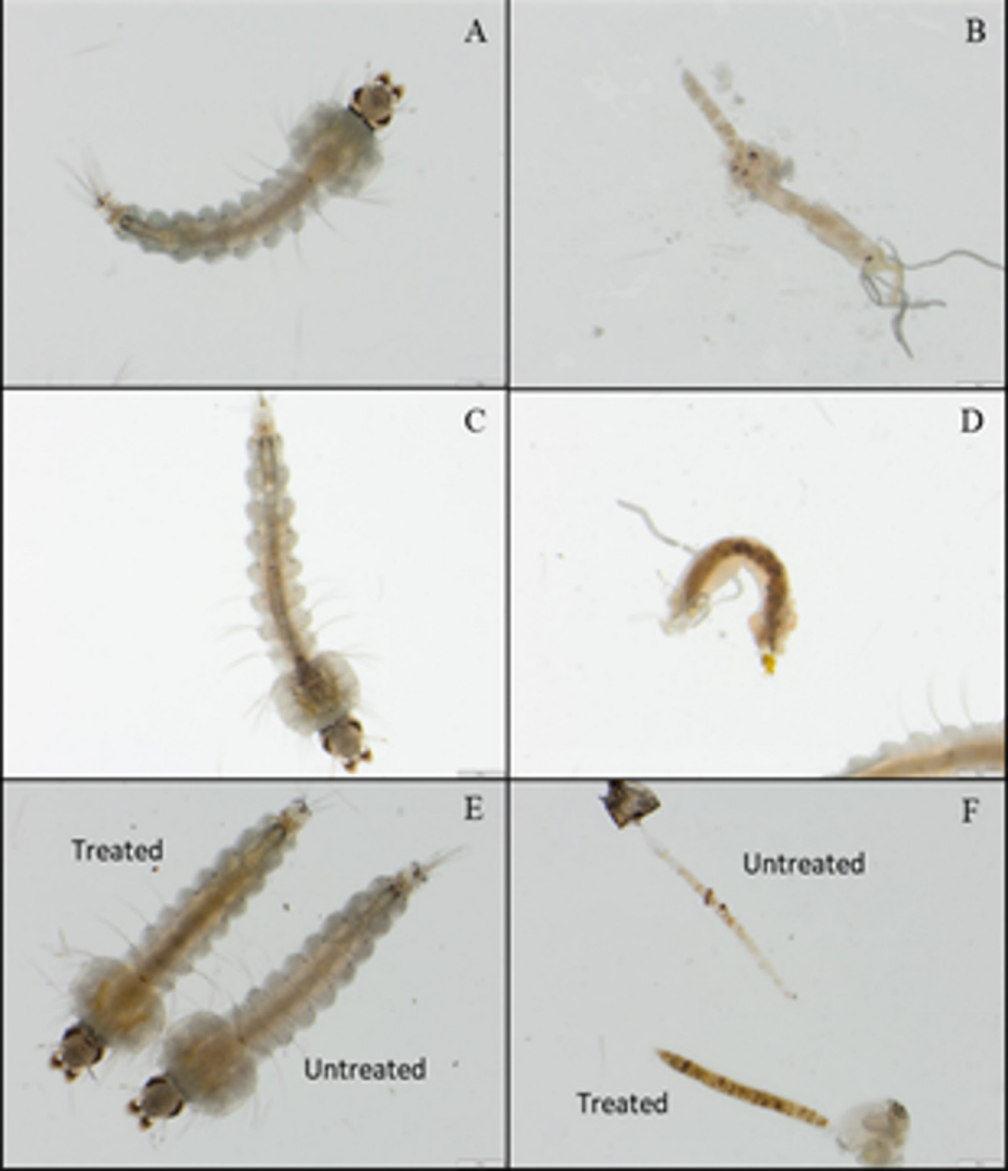
Fourth instar larvae reared in organic fertiliser and untreated conditions. Larvae were reared from hatchlings in either the OF-polluted of unpolluted conditions. Prior to pupation, larvae were examined and their midguts dissected. A: whole larvae reared under untreated control conditions. B: midgut of larvae reared under clean water conditions. C: whole larvae reared in organic fertiliser treated water. D: midgut of larvae reared in organic fertiliser treated water. E: comparison of untreated and treated larvae. F: comparison of untreated and treated midguts.

To determine whether any of the effects of observed were due to the consumption of the organic fertiliser, fourth instar larvae were examined for evidence of the material in their gut. Fig 6A and 6B represent a larvae reared in clean water and its’ resected midgut. There was no evidence of fertiliser in the food bolus. Fig 6C and 6D represents a larvae reared in organic fertiliser and its’ midgut respectively. There is evidence of organic fertiliser in the food bolus of the resected gut. Fig 6E shows a comparison of a treated and untreated 4^th^ instar larvae, and Fig 6F show the content of those larvae’s midguts, demonstrating the presence of organic fertiliser in the gut, suggesting that the larvae are ingesting the fertiliser.

## Discussion

The advantage conferred by larval exposure to organic fertiliser appears to be linked to the consumption of fertiliser by the larvae (Fig 6). This is suggested by the presence of the fertiliser in their guts. It has been reported that larval nutrition positively affects numerous life history traits, particularly in *An*. *arabiensis* [23, 39]. Although the effect of the consumption of organic detritus has been examined in container breeding mosquitoes such as *Aedes albopictus*, *Ae*. *aegytpi* and *Culex quinquefasciatus* [46–48], the effect is less clear in *Anopheles* mosquitoes. The aforementioned studies all examined organic detritus with particular reference to plant matter such as leaf litter, which would have a greater effect on *Aedes* mosquitoes than *Anopheles*.

A previous study demonstrated that the presence of cattle faeces enhanced the Anopheline population in a rice growing area under semi-field conditions [26]. The basis of this observation still remains unexplored. This study partially addresses this question. What is of interest about this exposure is that unlike exposure to inorganic or more toxic pollutants, insecticide susceptible as well as resistant mosquitoes gain an advantage. The advantages to insecticide susceptible strains are numerous, including shorter larval development time and increased adult longevity. Importantly, these observations were conserved across the member species of the *An*. *gambiae* complex assessed here. Insecticide resistant *An*. *arabiensis* seem to gain an advantage primarily in terms of increased insecticide tolerance. The effect of continued insecticide selection pressure appears to play a crucial role in this effect. While larval exposure also increases larval development rate, the size and longevity advantages seen in susceptible strains were not evident in the insecticide resistant, selected SENN-DDT strain. Although MBN-DDT was selected from MBN it retains its’ resistant phenotype despite the fact that selection has ceased. Therefore, it is representative of a strain that is no longer under selection pressure. This lack of difference in developmental rate between the two strains suggests a lack of fitness cost in the MBN-DDT strain. The increased rate of development in MBN mirrors the findings in SENN and SENN-DDT. This highlights the finding that insecticide susceptible strains gain a greater advantage in terms of increased development rate than insecticide resistant strains.

The marked difference between the advantages gained by insecticide resistant and susceptible strains is also observed in longevity. Like with larval development, insecticide susceptible strains gained a marked advantage in increased longevity. This may be due to nutritional advantages gained by the consumption of the organic fertiliser, and that the advantages in the insecticide susceptible stains are diffuse, resulting in increased rate of development, size (an indicator of fecundity [49, 50]) and longevity in insecticide susceptible strains. It is notable that a sex-specific difference in changes in longevity occurs in *An*. *merus*. Although there is no confirmation about why this may be so, it may be due to sex-specific preferences in the consumption of the organic fertiliser.

A previous study on the effect of nutrition of *An*. *arabiensis* insecticide resistance demonstrated that larval nutritional deprivation had a greater effect on the insecticide susceptible SENN strain than the resistant SENN-DDT strain [39]. These data, along with the findings of this study, suggested that the greatest advantage for the insecticide resistant, selected SENN-DDT strain was increased insecticide tolerance. This suggests that in this strain resource allocation is directed towards maintenance of the insecticide resistance phenotype, as metabolic resistance is an energetically costly process [51, 52].

The differences by which the MBN-DDT and SENN-DDT mediate increased insecticide tolerance are also worth noting. While the process appears to be mediated by increased cytochrome P450 activity in the MBN-DDT strain, general esterase activity was most markedly increased in the SENN-DDT strain. OF treatment significantly increased alpha-esterase activity in males, which may underlie the increase in male lethal time for both malathion and deltamethrin. The decreased deltamethrin lethal time is not explained by detoxification enzyme activity. Furthermore, the decrease in GST activity, coupled with a decreased catalase activity in the SENN-DDT strain, suggests a lowered requirement for oxidative stress defence, in turn suggesting a lowered oxidative stress burden in this strain, which is associated with increased insecticide resistance [53, 54]. This, however, would require measurement of oxidative stress markers.

Another mechanism whereby organic fertiliser pollution may be affecting insecticide resistant phenotypes is by the modulation of gut microflora. Larvae acquire their gut microbiome from the aquatic environment [55] and, therefore, the consumption of organic fertiliser may alter the gut bacterial composition. Gut bacterial composition has been demonstrated to play a crucial role in both life history and expression of insecticide resistance phenotypes [56].

The SENN and SENN-DDT strains have recently been used to test the effects of a number of stressors on the expression of insecticide resistance. It is worth noting that larval organic fertiliser caused the highest recorded increase in lethal time in SENN-DDT, more than herbicide or metal exposure [31, 32]. Crucially, this resulted in an increase in insecticide resistance intensity. Insecticide resistance intensity is one of the most crucial markers of the operational impact on vector control [42]. Therefore, the increase in deltamethrin and permethrin resistance intensity from low and moderate to high suggests that exposure to organic fertilizer at the larval stage can assist in producing resistance phenotypes at the adult stage that may have be operationally significant [35].

In conclusion, members of the *An*. *gambiae* complex are exposed to cattle manure during their immature aquatic stages. Using organic fertiliser as a proxy for cattle faeces, it is evident that mosquito larvae feed on the fertiliser which then confers numerous life history advantages (reduced larval development time, increase adult longevity, increased adult size, and increased insecticide tolerance and intensity), particularly for insecticide susceptible strains. The effect on increasing insecticide tolerance is most marked in insecticide resistant strains. The marked increase in insecticide tolerance and translation into an increase in pyrethroid resistance intensity in association with OF treatment, is predicted to produce a resistant phenotype that could exert an operational impact.

## Supporting information

S1 Table Supplementary Table 1: Average time to 50% mortality in all experimental strains. Significant differences from the control are highlighted in green. SENN-DDT males and females did not differ significantly from the control.
(DOCX)

## References

[1] Coetzee M, Hunt RH, Wilkerson R, Della Torre A, Coulibaly MB, Besansky NJ. *Anopheles coluzzii* and *Anopheles amharicus*, new members of the *Anopheles gambiae* complex. Zootaxa. 2013;3619:246–74. Epub 2013/01/01. . 26131476

[2] Sinka ME, Bangs MJ, Manguin S, Coetzee M, Mbogo CM, Hemingway J, et al The dominant *Anopheles* vectors of human malaria in Africa, Europe and the Middle East: occurrence data, distribution maps and bionomic precis. Parasites & vectors. 2010;3:117 10.1186/1756-3305-3-117 21129198 PMC3016360

[3] Gillies MT, Meillon BD. The Anophelinae of Africa south of the Sahara (Ethiopian Zoogeographical Region). Publications of the South African Institute for Medical Research 1968;54.

[4] Awolola TS, Oduola AO, Obansa JB, Chukwurar NJ, Unyimadu JP. *Anopheles gambiae* s.s. breeding in polluted water bodies in urban Lagos, southwestern Nigeria. J Vector Borne Dis. 2007;44(4):241–4. . 18092529

[5] Jones CM, Toe HK, Sanou A, Namountougou M, Hughes A, Diabate A, et al Additional selection for insecticide resistance in urban malaria vectors: DDT resistance in *Anopheles arabiensis* from Bobo-Dioulasso, Burkina Faso. PloS one. 2012;7(9):e45995 Epub 2012/10/11. 10.1371/journal.pone.0045995 PONE-D-12-13897 [pii]. 23049917 PMC3457957

[6] Poupardin R, Reynaud S, Strode C, Ranson H, Vontas J, David JP. Cross-induction of detoxification genes by environmental xenobiotics and insecticides in the mosquito *Aedes aegypti*: impact on larval tolerance to chemical insecticides. Insect Biochem Mol Biol. 2008;38(5):540–51. Epub 2008/04/15. S0965-1748(08)00023-4 [pii] 10.1016/j.ibmb.2008.01.004 . 18405832

[7] Poupardin R, Riaz MA, Jones CM, Chandor-Proust A, Reynaud S, David JP. Do pollutants affect insecticide-driven gene selection in mosquitoes? Experimental evidence from transcriptomics. Aquat Toxicol. 2012;114–115:49–57. Epub 2012/03/13. S0166-445X(12)00050-1 [pii] 10.1016/j.aquatox.2012.02.001 . 22406618

[8] Nkya TE, Akhouayri I, Kisinza W, David JP. Impact of environment on mosquito response to pyrethroid insecticides: facts, evidences and prospects. Insect Biochem Mol Biol. 2013;43(4):407–16. 10.1016/j.ibmb.2012.10.006 . 23123179

[9] Bara JJ, Montgomery A, Muturi EJ. Sublethal effects of atrazine and glyphosate on life history traits of *Aedes aegypti* and *Aedes albopictus* (Diptera: Culicidae). Parasitology research. 2014;113(8):2879–86. Epub 2014/05/24. 10.1007/s00436-014-3949-y . 24853538

[10] Mireji PO, Keating J, Hassanali A, Mbogo CM, Nyambaka H, Kahindi S, et al Heavy metals in mosquito larval habitats in urban Kisumu and Malindi, Kenya, and their impact. Ecotoxicol Environ Saf. 2008;70(1):147–53. Epub 2007/05/29 z. 10.1016/j.ecoenv.2007.03.012 ; PubMed Central PMCID: PMCPmc2673497. 17532467 PMC2673497

[11] Darriet F, Rossignol M, Chandre F. The combination of NPK fertilizer and deltamethrin insecticide favors the proliferation of pyrethroid-resistant *Anopheles gambiae* (Diptera: Culicidae). Parasite (Paris, France). 2012;19(2):159–64. Epub 2012/05/03. P3V192R7 [pii]. 10.1051/parasite/2012192159 22550627 PMC3671440

[12] Mutero CM, Ng'ang'a PN, Wekoyela P, Githure J, Konradsen F. Ammonium sulphate fertiliser increases larval populations of *Anopheles arabiensis* and culicine mosquitoes in rice fields. Acta tropica. 2004;89(2):187–92. Epub 2004/01/21. . 14732240 10.1016/j.actatropica.2003.08.006

[13] Mahande A, Mosha F, Mahande J, Kweka E. Feeding and resting behaviour of malaria vector, *Anopheles arabiensis* with reference to zooprophylaxis. Malaria journal. 2007;6(1):100 10.1186/1475-2875-6-100 17663787 PMC1964787

[14] Kitau J, Oxborough RM, Tungu PK, Matowo J, Malima RC, Magesa SM, et al Species shifts in the *Anopheles gambiae* complex: do LLINs successfully control *Anopheles arabiensis*? PloS one. 2012;7(3):e31481 10.1371/journal.pone.0031481 22438864 PMC3306310

[15] Killeen GF. Characterizing, controlling and eliminating residual malaria transmission. Malaria journal. 2014;13:330 Epub 2014/08/26. 1475-2875-13-330 [pii] 10.1186/1475-2875-13-330 25149656 PMC4159526

[16] Dandalo LC, Brooke BD, Munhenga G, Lobb LN, Zikhali J, Ngxongo SP, et al Population Dynamics and *Plasmodium falciparum* (Haemosporida: Plasmodiidae) Infectivity Rates for the Malaria Vector *Anopheles arabiensis* (Diptera: Culicidae) at Mamfene, KwaZulu-Natal, South Africa. Journal of medical entomology. 2017;54(6):1758–66. Epub 2017/10/03. 10.1093/jme/tjx169 . 28968846

[17] Hargreaves K, Hunt RH, Brooke BD, Mthembu J, Weeto MM, Awolola TS, et al *Anopheles arabiensis* and *An*. *quadriannulatus* resistance to DDT in South Africa. Med Vet Entomol. 2003;17(4):417–22. Epub 2003/12/04. . 14651656 10.1111/j.1365-2915.2003.00460.x

[18] Sharp BL, Le Sueur D, Wilken GB, Bredenkamp BL, Ngxongo S, Gouws E. Assessment of the residual efficacy of lambda-cyhalothrin. 2. A comparison with DDT for the intradomiciliary control of *Anopheles arabiensis* in South Africa. Journal of the American Mosquito Control Association. 1993;9(4):414–20. Epub 1993/12/01. . 8126475

[19] Githeko AK, Service MW, Mbogo CM, Atieli FK. Resting behaviour, ecology and genetics of malaria vectors in large scale agricultural areas of Western Kenya. Parassitologia. 1996;38(3):481–9. . 9257337

[20] Ijumba JN, Lindsay SW. Impact of irrigation on malaria in Africa: paddies paradox. Med Vet Entomol. 2001;15(1):1–11. . 11297093 10.1046/j.1365-2915.2001.00279.x

[21] Mwangangi JM, Muturi EJ, Shililu J, Muriu SM, Jacob B, Kabiru EW, et al Survival of immature *Anopheles arabiensis* (Diptera: Culicidae) in aquatic habitats in Mwea rice irrigation scheme, central Kenya. Malaria journal. 2006;5:114 Epub 2006/11/28. 10.1186/1475-2875-5-114 ; PubMed Central PMCID: PMCPmc1698490. 17125501 PMC1698490

[22] Mwangangi J, Shililu J, Muturi E, Gu W, Mbogo C, Kabiru E, et al Dynamics of immature stages of *Anopheles arabiensis* and other mosquito species (Diptera: Culicidae) in relation to rice cropping in a rice agro-ecosystem in Kenya. J Vector Ecol. 2006;31(2):245–51. . 17249341 10.3376/1081-1710(2006)31[245:doisoa]2.0.co;2

[23] Ye-Ebiyo Y, Pollack RJ, Spielman A. Enhanced development in nature of larval *Anopheles arabiensis* mosquitoes feeding on maize pollen. Am J Trop Med Hyg. 2000;63(1–2):90–3. . 11358003 10.4269/ajtmh.2000.63.90

[24] Mutero CM, Kabutha C, Kimani V, Kabuage L, Gitau G, Ssennyonga J, et al A transdisciplinary perspective on the links between malaria and agroecosystems in Kenya. Acta tropica. 2004;89(2):171–86. Epub 2004/01/21. . 14732239 10.1016/j.actatropica.2003.07.003

[25] Seyoum A, Balcha F, Balkew M, Ali A, Gebre-Michael T. Impact of cattle keeping on human biting rate of anopheline mosquitoes and malaria transmission around Ziway, Ethiopia. East African medical journal. 2002;79(9):485–90. Epub 2003/03/11. . 12625690 10.4314/eamj.v79i9.9121

[26] Jarju LB, Fillinger U, Green C, Louca V, Majambere S, Lindsay SW. Agriculture and the promotion of insect pests: rice cultivation in river floodplains and malaria vectors in The Gambia. Malaria journal. 2009;8:170 Epub 2009/07/29. 10.1186/1475-2875-8-170 ; PubMed Central PMCID: PMCPmc2734858. 19635125 PMC2734858

[27] Mbokazi F, Coetzee M, Brooke B, Govere J, Reid A, Owiti P, et al Changing distribution and abundance of the malaria vector *Anopheles merus* in Mpumalanga Province, South Africa. Public health action. 2018;8(Suppl 1):S39–S43. Epub 04/25. 10.5588/pha.17.0034 . 29713593 PMC5912421

[28] Alam MS, Al-Amin HM, Elahi R, Chakma S, Kafi MAH, Khan WA, et al Abundance and Dynamics of *Anopheles* (Diptera: Culicidae) Larvae in a Malaria Endemic Area of Bangladesh. Journal of medical entomology. 2018;55(2):382–91. Epub 2017/11/29. 10.1093/jme/tjx196 . 29182782

[29] Shapiro LL, Murdock CC, Jacobs GR, Thomas RJ, Thomas MB. Larval food quantity affects the capacity of adult mosquitoes to transmit human malaria. Proceedings Biological sciences. 2016;283(1834). Epub 2016/07/15. 10.1098/rspb.2016.0298 ; PubMed Central PMCID: PMCPmc4947883. 27412284 PMC4947883

[30] Kibuthu TW, Njenga SM, Mbugua AK, Muturi EJ. Agricultural chemicals: life changer for mosquito vectors in agricultural landscapes? Parasites & vectors. 2016;9:500 Epub 2016/09/15. 10.1186/s13071-016-1788-7 ; PubMed Central PMCID: PMCPmc5022241. 27624456 PMC5022241

[31] Oliver SV, Brooke BD. The effect of commercial herbicide exposure on the life history and insecticide resistance phenotypes of the major malaria vector *Anopheles arabiensis* (Diptera: culicidae). Acta tropica. 2018;188:152–60. Epub 2018/09/05. 10.1016/j.actatropica.2018.08.030 . 30179608

[32] Oliver SV, Brooke BD. The effect of metal pollution on the life history and insecticide resistance phenotype of the major malaria vector *Anopheles arabiensis* (Diptera: Culicidae). PloS one. 2018;13(2):e0192551 Epub 2018/02/07. 10.1371/journal.pone.0192551 ; PubMed Central PMCID: PMCPmc5800662. 29408922 PMC5800662

[33] Mireji PO, Keating J, Hassanali A, Mbogo CM, Muturi MN, Githure JI, et al Biological cost of tolerance to heavy metals in the mosquito *Anopheles gambiae*. Med Vet Entomol. 2010;24(2):101–7. Epub 2010/04/09. 10.1111/j.1365-2915.2010.00863.x ; PubMed Central PMCID: PMCPmc2921613. 20374478 PMC2921613

[34] Belz RG, Duke SO. Herbicides and plant hormesis. Pest management science. 2014;70(5):698–707. Epub 2014/01/22. 10.1002/ps.3726 . 24446388

[35] Brito IP, Tropaldi L, Carbonari CA, Velini ED. Hormetic effects of glyphosate on plants. Pest management science. 2018;74(5):1064–70. Epub 2017/01/18. 10.1002/ps.4523 . 28094904

[36] Bahgat IM. Impact of physical and chemical characteristics of breeding sites on mosquito larval abundance at Ismailia Governorate, Egypt. Journal of the Egyptian Society of Parasitology. 2013;43(2):399–406. Epub 2013/11/23. . 24260817 10.12816/0006395

[37] Baker SL, Yan ND. Accumulated organic debris in catch basins improves the efficacy of S-methoprene against mosquitoes in Toronto, Ontario, Canada. Journal of the American Mosquito Control Association. 2010;26(2):172–82. Epub 2010/07/24. 10.2987/09-5928.1 . 20649127

[38] Hunt RH, Brooke BD, Pillay C, Koekemoer LL, Coetzee M. Laboratory selection for and characteristics of pyrethroid resistance in the malaria vector Anopheles funestus. Med Vet Entomol. 2005;19(3):271–5. 10.1111/j.1365-2915.2005.00574.x . 16134975

[39] Oliver SV, Brooke BD. The effect of larval nutritional deprivation on the life history and DDT resistance phenotype in laboratory strains of the malaria vector *Anopheles arabiensis*. Malaria journal. 2013;12:44 Epub 2013/02/02. 10.1186/1475-2875-12-44 ; PubMed Central PMCID: PMCPmc3570311. 23368928 PMC3570311

[40] Oliver SV, Brooke BD. The effect of multiple blood-feeding on the longevity and insecticide resistant phenotype in the major malaria vector *Anopheles arabiensis* (Diptera: Culicidae). Parasites & vectors. 2014;7:390 Epub 2014/08/26. 10.1186/1756-3305-7-390 ; PubMed Central PMCID: PMCPmc4161849. 25150975 PMC4161849

[41] Oliver SV, Brooke BD. The effect of elevated temperatures on the life history and insecticide resistance phenotype of the major malaria vector *Anopheles arabiensis* (Diptera: Culicidae). Malaria journal. 2017;16(1):73 Epub 2017/02/15. 10.1186/s12936-017-1720-4 ; PubMed Central PMCID: PMCPmc5307775. 28193292 PMC5307775

[42] Venter N, Oliver SV, Muleba M, Davies C, Hunt RH, Koekemoer LL, et al Benchmarking insecticide resistance intensity bioassays for *Anopheles* malaria vector species against resistance phenotypes of known epidemiological significance. Parasites & vectors. 2017;10(1):198 Epub 2017/04/22. 10.1186/s13071-017-2134-4 ; PubMed Central PMCID: PMCPmc5397746. 28427447 PMC5397746

[43] Nardini L, Christian RN, Coetzer N, Koekemoer LL. DDT and pyrethroid resistance in *Anopheles arabiensis* from South Africa. Parasites & vectors. 2013;6(1):229 Epub 2013/08/09. 10.1186/1756-3305-6-229 ; PubMed Central PMCID: PMCPmc3751093. 23924547 PMC3751093

[44] Lyimo EO, Takken W. Effects of adult body size on fecundity and the pre-gravid rate of *Anopheles gambiae* females in Tanzania. Med Vet Entomol. 1993;7(4):328–32. Epub 1993/10/01. . 8268486 10.1111/j.1365-2915.1993.tb00700.x

[45] WHO. Test procedures for insecticide resistance monitoring in malaria vector mosquitoes. http://wwwwhoint/iris/handle/10665/250677 2016.

[46] Allgood DW, Yee DA. Influence of resource levels, organic compounds and laboratory colonization on interspecific competition between the Asian tiger mosquito *Aedes albopictus* (Stegomyia albopicta) and the southern house mosquito *Culex quinquefasciatus*. Med Vet Entomol. 2014;28(3):273–86. Epub 2014/01/22. 10.1111/mve.12047 ; PubMed Central PMCID: PMCPmc4105337. 24444185 PMC4105337

[47] Murrell EG, Damal K, Lounibos LP, Juliano SA. Distributions of Competing Container Mosquitoes Depend on Detritus Types, Nutrient Ratios, and Food Availability. Annals of the Entomological Society of America. 2011;104(4):688–98. Epub 2012/06/19. 10.1603/AN10158 ; PubMed Central PMCID: PMCPmc3375989. 22707761 PMC3375989

[48] Ponnusamy L, Wesson DM, Arellano C, Schal C, Apperson CS. Species composition of bacterial communities influences attraction of mosquitoes to experimental plant infusions. Microbial ecology. 2010;59(1):158–73. Epub 2009/07/31. 10.1007/s00248-009-9565-1 ; PubMed Central PMCID: PMCPmc4561554. 19641948 PMC4561554

[49] Armbruster P, Hutchinson RA. Pupal mass and wing length as indicators of fecundity in *Aedes albopictus* and *Aedes* geniculatus (Diptera: Culicidae). Journal of medical entomology. 2002;39(4):699–704. Epub 2002/07/30. . 12144308 10.1603/0022-2585-39.4.699

[50] Blackmore MS, Lord CC. The relationship between size and fecundity in *Aedes albopictus*. J Vector Ecol. 2000;25(2):212–7. Epub 2001/02/24. . 11217219

[51] Diniz DF, de Melo-Santos MA, Santos EM, Beserra EB, Helvecio E, de Carvalho-Leandro D, et al Fitness cost in field and laboratory Aedes aegypti populations associated with resistance to the insecticide temephos. Parasites & vectors. 2015;8:662 Epub 2015/12/31. 10.1186/s13071-015-1276-5 ; PubMed Central PMCID: PMCPmc4696322. 26715037 PMC4696322

[52] Rivero A, Magaud A, Nicot A, Vezilier J. Energetic cost of insecticide resistance in *Culex pipiens* mosquitoes. Journal of medical entomology. 2011;48(3):694–700. Epub 2011/06/15. . 21661333 10.1603/me10121

[53] Champion CJ, Xu J. Redox state affects fecundity and insecticide susceptibility in *Anopheles gambiae*. Scientific reports. 2018;8(1):13054 Epub 2018/08/31. 10.1038/s41598-018-31360-2 ; PubMed Central PMCID: PMCPmc6115382. 30158658 PMC6115382

[54] Oliver SV, Brooke BD. The Role of Oxidative Stress in the Longevity and Insecticide Resistance Phenotype of the Major Malaria Vectors *Anopheles arabiensis* and *Anopheles funestus*. PloS one. 2016;11(3):e0151049 Epub 2016/03/11. 10.1371/journal.pone.0151049 ; PubMed Central PMCID: PMCPmc4786153. 26964046 PMC4786153

[55] Coon KL, Vogel KJ, Brown MR, Strand MR. Mosquitoes rely on their gut microbiota for development. Molecular ecology. 2014;23(11):2727–39. Epub 2014/04/29. 10.1111/mec.12771 ; PubMed Central PMCID: PMCPmc4083365. 24766707 PMC4083365

[56] Dada N, Sheth M, Liebman K, Pinto J, Lenhart A. Whole metagenome sequencing reveals links between mosquito microbiota and insecticide resistance in malaria vectors. Scientific reports. 2018;8(1):2084 Epub 2018/02/03. 10.1038/s41598-018-20367-4 ; PubMed Central PMCID: PMCPmc5794770. 29391526 PMC5794770

