# Supplementary material for: The effects of larval organic fertiliser exposure on the larval development, adult longevity and insecticide tolerance of zoophilic members of the Anopheles gambiae complex (Diptera: Culicidae)
Source: PLoS One. 2019 Apr 18;14(4):e0215552. doi: 10.1371/journal.pone.0215552 (PMC6472872; doi:10.1371/journal.pone.0215552)
Supplement: S1 Table — Significant differences from the control are highlighted in green. SENN-DDT males and females did not differ significantly from the control. (DOCX) [file pone.0215552.s001.docx]

**Supplementary table 1: Average time to 50% mortality in all experimental strains.** Significant differences from the control are highlighted in green. SENN-DDT males and females did not differ significantly from the control.

|  | **Control** |  | **Treatment** | |
| --- | --- | --- | --- | --- |
| **Strain** | **Male** | **Female** | **Male** | **Female** |
| SENN (*An. arabiensis*) | 31(0.3;0.6) | 29(0.2;0.5) | 34(0.3;0.6) | 32(0.3;0.6) |
| SENN-DDT (*An. arabiensis*) | 28(0.2;0.5) | 26(0.3;0.5) | 29(0.3;0.4) | 28(0.2;0.5) |
| MBN (*An. arabiensis*) | 17(0.1;0.5) | 26(0.3;0.7) | 29(0.2;0.6) | 32(0.2;0.6) |
| MBN-DDT (*An. arabiensis*) | 24(0.2;0.6) | 24(0.2;0.6) | 28(0.1;0.4) | 36(0.1;0.5) |
| MAFUS (*An. merus*) | 22(0.3;0.6) | 16(0.2;0.6) | 31(0.2;0.6) | 18(0.2;0.6) |
| SAGWE (*An. quadriannulatus*) | 20(0.3;0.7) | 23(0.2;0.6) | 31 (0.2;0.7) | 35(0.2;0.6) |
| Indicates a significant increase from the control | | |  |  |
